# A simple, fast, and accurate method of phylogenomic inference

**DOI:** 10.1186/gb-2008-9-10-r151

**Published:** 2008-10-13

**Authors:** Martin Wu, Jonathan A Eisen

**Affiliations:** 1 Genome Center, University of California, One Shields Avenue, Davis, CA 95616, USA; 2 Section of Evolution and Ecology, College of Biological Sciences, University of California, One Shields Avenue, Davis, CA 95616, USA; 3 Department of Medical Microbiology and Immunology, School of Medicine, University of California, One Shields Avenue, Davis, CA 95616, USA

## Abstract

The explosive growth of genomic data provides an opportunity to make increased use of protein markers for phylogenetic inference. We have developed an automated pipeline for phylogenomic analysis (AMPHORA) that overcomes the existing bottlenecks limiting large-scale protein phylogenetic inference. We demonstrated its high throughput capabilities and high quality results by constructing a genome tree of 578 bacterial species and by assigning phylotypes to 18,607 protein markers identified in metagenomic data collected from the Sargasso Sea.

## Abstract 2

An automated pipeline for phylogenomic analysis (AMPHORA) is presented that overcomes existing limits to large-scale protein phylogenetic inference.

## Background

Since the 1970s the use of small subunit (SSU) rRNA (SSU rRNA) sequences has revolutionized microbial classification, systematics, and ecology. The SSU rRNA gene has become the most sequenced gene, with hundreds of thousands of its sequences now deposited in public databases. It has become the current 'gold standard' in microbial diversity studies, and for good reasons. For one, it is present in all microbial organisms. For another, the gene sequence is highly conserved at both ends. This enables one to obtain nearly full-length SSU rRNA gene sequences by polymerase chain reaction amplification using 'universal' primers and without having to isolate and culture the organism in question. Until very recently, the vast majority of microbes were identified and classified only by recovering and sequencing their SSU rRNA genes. This single sequence of approximately 1.5 kilobases is often the only information we have about the organism from which it came - the only way we know that it exists in the natural environment.

Although the SSU rRNA gene has been extremely valuable for phylogenetic studies, it has its limitations. For example, it has been well documented that evolutionarily distant SSU rRNA genes that are similar in nucleotide composition have been consistently - but nevertheless incorrectly - placed close together in phylogenetic trees [1,2,1]. Furthermore, inferring the phylogeny of organisms from any single gene carries some risks and must be corroborated by the use of other phylogenetic markers. Many researchers turned to protein encoding genes such as *EF-Tu*, *rpoB*, *recA*, and *HSP70 *[3]. Because protein sequences are conserved at the amino acid level instead of at the nucleotide level, phylogenetic analyses of protein sequences are in general less prone to the nucleotide compositional bias seen in SSU rRNA [2,4-6]. In addition, the less constrained variation at the third codon position allows these genes to be used in studies of more closely related organisms. However, because of difficulties in cloning protein encoding genes from diverse species, SSU rRNA remained the gold standard.

The situation changed with the advent of genomic sequencing. Each complete genome sequence brings with it the sequences for all protein encoding genes in that organism. Now, not only can one build gene trees based on a favorite protein encoding gene, but also one has the option to concatenate multiple gene sequences to construct trees on the 'genome level'. Possessing more phylogenetic signals, such 'genome trees' or 'super-matrix trees' are less susceptible to the stochastic errors than those built from a single gene [7]. Recent studies attempting to reconstruct the tree of life have demonstrated the power of this approach [8,9] (for review [10]). Likewise, genome trees have also been used successfully to reassess the phylogenetic positions of individual species [11,12]. It is worth pointing out, however, that the genome trees are still susceptible to systematic errors caused by compositional biases, unrealistic evolutionary models, and inadequate taxonomic sampling [7,13,14].

Despite its demonstrated usefulness, phylogenetic inference based on protein markers has been limited in application, mainly because of the formidable technical difficulties inherent in this approach. Typically, molecular phylogenetic inference involves three steps: retrieval of homologous sequences, creation of multiple sequence alignments, and phylogenetic tree construction. Because only characters of common ancestry can be used to infer the evolutionary history, the most critical step is sequence alignment, in which sequences are overlaid horizontally on each other in such a way that, ideally, each column in the alignment would only contain homologous characters (amino acids or nucleotides). To ensure this positional homology, the alignments must be curated - a process that evaluates the probable homology of each column or position in the alignments.

Positions for which the assignment of homology is uncertain are then excluded from further analysis by masking [15]. Judicious masking increases the signal-to-noise ratio and often improves the discriminatory power of the phylogenetic methods [16]. Unfortunately, curation requires skilled manual intervention, thus making it impractical to process suitably the massive amount of genome sequence data now available. Frequently, masking is simply ignored. Automated masking to remove alignment positions that contain gaps or that have a low degree of conservation has not been satisfactory. For example, using these criteria and given a set of *ad hoc *parameters such as the minimum block length, GBLOCKS automatically selects conserved blocks from multiple sequence alignments for phylogenetic analysis. However, trees constructed using GBLOCKS-treated alignments have been found to have dramatically weaker support, possibly because of excessive removal of informative sites by the program [17]. In addition, although many programs are available to automate the creation of multiple sequence alignments, their use for the *de novo *alignment of a large protein family is still fairly time consuming.

To overcome these problems, we have developed an automated pipeline for building concatenated genome trees using multiple protein markers, thus making this powerful method applicable on a larger scale. Our pipeline can rapidly and accurately generate highly reproducible multiple sequence alignments for a set of selected phylogenetic markers. More importantly, unlike previous automated methods [9] it can mask the alignments with quality equivalent to that of curation by humans.

The same pipeline can also be applied to metagenomic data analyses. In metagenomics or environmental genomics, natural populations of microbes are collected from the environment; their DNAs are cloned and directly sequenced. One fundamental goal of metagenomics is to determine who is present in the community and what they are doing. Phylogenetic analysis of markers present in these collected samples can be very informative in revealing who is there. If the marker happens to be part of a larger assembled sequence fragment, then the entire fragment can be anchored by that marker to a specific taxonomic clade. In this way, environmental shotgun sequences can be sorted into taxon-specific 'bins' *in silico*, thereby allowing us to determine who is doing what.

The most striking finding to date from this approach was the discovery of a proteorhodopsin gene in bacteria, a homolog of the bacteriorhodopsin gene previously found only in some archaea. In this case, the gene could be anchored within the bacteria because it was found to be associated with a bacterial SSU rRNA gene [18]. However, because the SSU rRNA gene constitutes only a tiny fraction of any genome, the probability that any given sequence fragment can be anchored to a specific taxonomic clade by using this one gene is small. Thus, phylotyping of metagenomic data can greatly benefit from the use of alternative phylogenetic markers such as the multiple protein markers described below.

In this paper, we introduce AMPHORA (a pipeline for AutoMated PHylogenOmic infeRence) and demonstrate two significant applications: building a genome tree from 578 complete bacterial genomes that are available at the time of the study and identifying bacterial phylotypes from metagenomic data collected from the Sargasso Sea.

## Results and discussion

### The AMPHORA pipeline

#### Introduction

**Figure 1:**
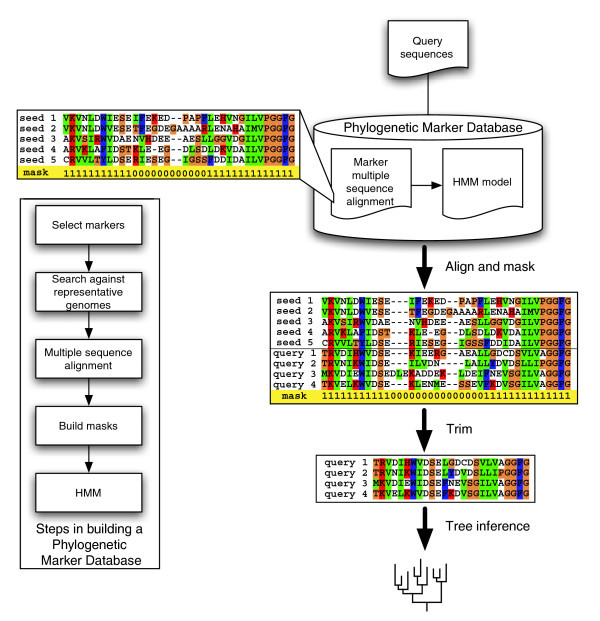
A flowchart illustrating the major components of AMPHORA. The marker protein sequences from representative genomes are retrieved, aligned, and masked. Profile hidden Markov models (HMMs) are then built from those 'seed' alignments. New sequences of interest are rapidly and accurately aligned to the trusted seed alignments through HMMs. Predefined masks embedded within the 'seed' alignment are then applied to trim off regions of ambiguity before phylogenetic inference. Alignment columns marked with '1' or '0' were included or excluded, respectively, during further phylogenetic analysis.

With the rapid increase in available genomic sequence data, there is an ever-urgent need for automated phylogenetic analyses using protein sequences. However, automation is frequently accompanied by reduced quality. We introduce here a fully automated method that is not only fast but also is of high quality. The main components of our approach are shown in Figure 1, and their implementation is described in detail in the Material and methods section (below). Designed to align and trim protein sequences rapidly, reliably, and reproducibly, AMPHORA eliminates one of the tightest bottlenecks in large-scale protein phylogenetic inference. It can be used for phylogenetic analyses of single genes or whole genomes.

#### Protein phylogenetic marker database

The core of AMPHORA is a protein phylogenetic marker database that contains curated protein sequence alignments with trimming masks and corresponding profile hidden Markov models (HMMs). Thirty-one protein encoding phylogenetic marker genes (*dnaG*, *frr*, *infC*, *nusA*, *pgk*, *pyrG*, *rplA*, *rplB*, *rplC*, *rplD*, *rplE*, *rplF*, *rplK*, *rplL*, *rplM*, *rplN*, *rplP*, *rplS*, *rplT*, *rpmA*, *rpoB*, *rpsB*, *rpsC*, *rpsE*, *rpsI*, *rpsJ*, *rpsK*, *rpsM*, *rpsS*, *smpB*, and *tsf*) from representatives of complete bacterial genomes were individually aligned using CLUSTALW. The alignments were curated and trimming masks were added manually by visually inspecting the alignments. We selected these proteins because they are universally distributed in bacteria; the vast majority of them exist as single copy genes within each genome; and they are housekeeping genes that are involved in information processing (replication, transcription, and translation) or central metabolism, and thus are thought to be relatively recalcitrant to lateral gene transfer [19].

#### High quality and highly reproducible sequence alignments

Molecular phylogenetic inference assumes common ancestry, or homology, for every single column of a multiple sequence alignment. When this assumption is violated, phylogenetic signal can be obscured by noise. It has been shown that alignment quality can have greater impact on the final tree than does the tree building method employed [20]. Therefore, preparing high quality sequence alignments is a most critical part of any molecular phylogenetic analysis. This preparation typically involves careful but tedious manual editing and trimming of the generated alignments, and thus remains the greatest challenge to automation. When scaling up this process, the trimming step is often simply ignored. Automated trimming based on the number of gaps in each column or each column's conservation score can be used to select conserved blocks, but this still is not satisfactory when a high quality tree is required [17].

We overcame this problem by taking advantage of a unique feature of profile HMM-based multiple sequence alignments. When using HMMs to align sequences, new sequences can be mapped back, residue by residue, onto the 'seed' alignment from which that HMM originated. When the seed alignment includes an accurate human curated mask, the newly generated alignments can be automatically trimmed accordingly, thus producing high quality alignments without requiring further human intervention. In addition, the HMM model is the only variable in this automated alignment and trimming. When the same model is used, the alignments generated thereby are completely additive and reproducible, thus enabling meaningful comparison of the results from different phylogenetic studies or different researchers.

#### Speed

Another big advantage of using an HMM-based approach is speed. For example, AMPHORA needs only 0.5 minutes on an average desktop computer (Intel Pentium CPU 3.2 GHz) to align 340 sequences of the rpoB family. In comparison, the same job takes *de novo *pair-wise alignment methods such as CLUSTALW and MUSCLE 120 and 12 minutes, respectively. This is because our HMM-based method aligns sequences by comparing them only once each to the HMM model. As a result, the computational cost increases linearly with the number of sequences to be aligned. In contrast, the computational cost of a pair-wise alignment approach increases polynomially and can soon become prohibitively expensive.

### Application I: Bacterial genome trees

#### Constructing a 'genome' tree

**Figure 2:**
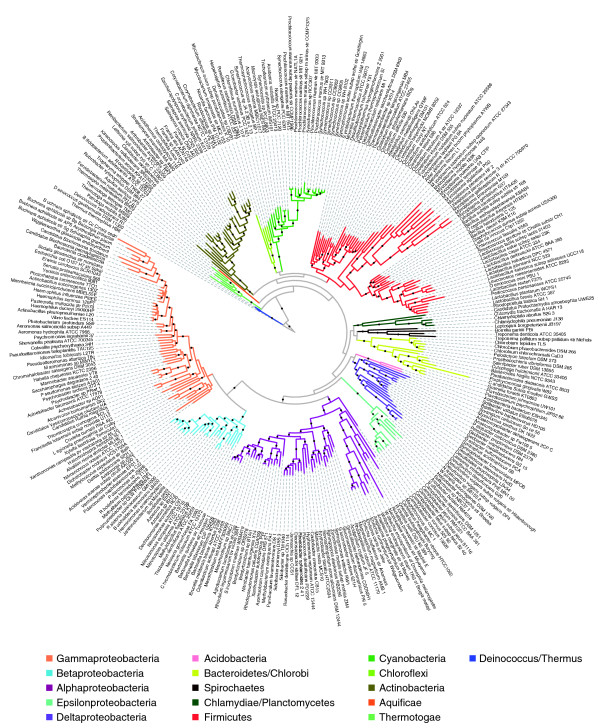
An unrooted maximum likelihood bacterial genome tree. The tree was constructed from concatenated protein sequence alignments derived from 31 housekeeping genes. All major phyla are separated into their monophyletic groups and are highlighted by color. The branches with bootstrap support of over 80 (out of 100 replicates) are indicated with black dots. Although the relationships among the phyla are not strongly supported, those below the phylum level show very respectable support. The radial tree was generated using iTOL [42].

We downloaded 578 complete bacterial genomes available at the time of our study from the National Center for Biotechnology Information (NCBI) RefSeq collections (Additional data file 1). Protein marker sequences for 31 proteins were retrieved, aligned, trimmed, and concatenated as described in the Materials and methods section (see below). This resulted in a mega-alignment of 5,591 good amino acid positions (columns) by 578 species (rows). A maximum likelihood genome tree was constructed from this mega-alignment (Additional data file 2). A bootstrapped maximum likelihood genome tree of 310 representatives is shown in Figure 2.

As with trees built from SSU rRNA data, all of the major bacterial phyla are well separated into their own monophyletic groups, even though the relationships among some of them remain unclear. Strikingly, unlike the SSU rRNA tree, the bushy area (intermediate levels) of our tree is highly resolved. In the γ-proteobacteria, for example, the nodes separating taxa into different orders, families, and genera receive generally excellent bootstrapping support, whereas uncertainty is high in the corresponding regions of the SSU rRNA tree (Additional data file 3). Highly robust organismal phylogenies of γ-proteobacteria and α-proteobacteria have been inferred previously using hundreds of commonly shared genes [21,22] and are congruent to our genome tree. This reflects the much-reduced stochastic noise present in the concatenated protein sequences compared with that of a single, slowly evolving SSU rRNA gene. This uncertainty in the SSU rRNA tree - the backbone of modern microbial systematics - often prevents microbial taxonomists from placing new species or genera within higher taxa, particularly at these intermediate levels [23]. When such assignments were nevertheless made for these problematic taxa, inconsistency was introduced into the taxonomic nomenclature. For example, taxa assigned to the orders *Alteromondales*, *Pseudomonadales*, and *Oceanospirillales *in Bergey's Taxonomic Outline of Prokaryotes [23] are intermingled and paraphyletic in our genome tree. It is our view that the taxonomy needs to be revisited and possibly revised in such cases.

#### Genome-based microbial taxonomy

Although use of SSU rRNA was a landmark advancement in molecular microbial systematics, genome sequences provide an important alternative and complement [11,12]. Phylogenetic trees built from multiple genes are more robust in resolving taxonomic relationships below the phylum level and hence provide an excellent alternative phylogenetic framework for microbial systematics. Until many more genomes have been sequenced, however, a hybrid approach may be most fruitful. A genome tree built from sequenced genomes can be used as a scaffold; species for which we lack full genome sequences can be placed by comparing their SSU rRNA sequences with those of sequenced species.

#### Average rate of protein evolution in bacterial genomes

The average rates of protein evolution are proportional to the branch lengths of our genome tree. The branch length varies widely among different lineages. For example, as has been previously reported, bacteria that have adopted an intracellular lifestyle have, in general, evolved more rapidly [24], with *Wigglesworthia glossinidia *(the endosymbiont of *Glossina brevipalpis*) and *Neorickettsia sennetsu str. Miyayama *evolving at the fastest pace. The slowest rates are found in a group of spore forming bacteria such as *Carboxydothermus hydrogenoformans*, *Moorella thermoacetica*, *Clostridium *spp., and *Bacillus *spp. These slow rates are of particular interest because it has been suggested that they might be related to the longer generation times for organisms that spend a significant fraction of their time as dormant spores. Our data for spore forming bacteria are consistent with that hypothesis and differ strikingly from the findings of a recent study [25], which identified no generation time effect in these organisms.

### Application II: metagenomic phylotyping

#### Reanalysis of the phylotypes reported in the Sargasso Sea

**Figure 3:**
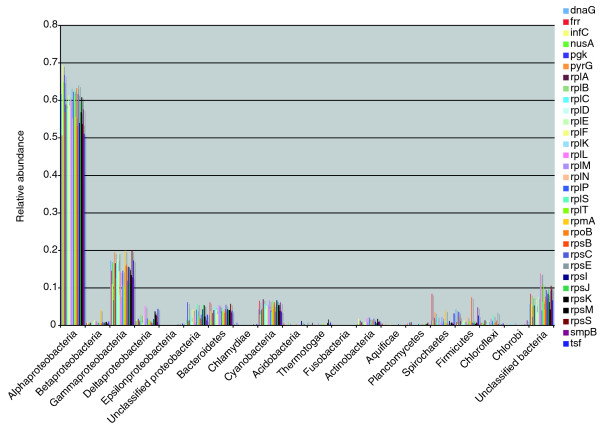
Major phylotypes identified in Sargasso Sea metagenomic data. The metagenomic data previously obtained from the Sargasso Sea was reanalyzed using AMPHORA and the 31 protein phylogenetic markers. The microbial diversity profiles obtained from individual markers are remarkably consistent. The breakdown of the phylotyping assignments by markers and major taxonomic groups is listed in Additional data file 5.

We used our automated pipeline to reanalyze the environmental shotgun sequencing data collected from the Sargasso Sea and phylotyped in a previous study [26]. The approximately 1.1 million predicted genes yielded a total of 18,607 genes that corresponded to our 31 protein markers and that were long enough for phylogenetic analysis. Figure 3 illustrates the distribution of each of the 31 protein markers among the major phylotypes. Our analysis identifies the α-proteobacteria as the most abundant group, because more than half of the marker sequences were assigned to this group. Notably, the various individual protein markers present remarkably consistent microbial diversity profiles, thus suggesting that results for different markers may be additive.

It was noted that SSU rRNA gives significantly different estimates of microbial composition than those by protein markers [26]. This is believed to be caused by large variations in rRNA gene copy numbers among different species. The protein markers used in our study are nearly all single-copy genes and thus should, theoretically, give a more accurate estimation of the microbial composition. One factor affecting our analysis is that the peptides are based on assemblies rather than sequence reads. Therefore, our method will underestimate those organisms that have deep coverage in assemblies. This is one major reason why depth of coverage should be provided with metagenomics assemblies and annotation.

Members of the α-proteobacterial SAR11 clade are the most dominant micro-organisms in the Sargasso Sea [27]. At the time of the Sargasso Sea metagenomics study, there were no complete genome sequences available for members of the SAR11 clade, and thus many of the SAR11 sequences could not be anchored. The genome of one SAR11 member, namely *Candidatus Pelagibacter ubique*, was subsequently sequenced, which allows for much finer phylotyping now. In our phylotyping analyses, 8,656 marker sequences (46.5% of the total) form outgroups to only *P. ubique*. We have assigned them to the SAR11 clade because their closest neighbor in the tree is *P. ubique *and their dominance in the population is consistent with previous quantitative estimations by fluoresence *in situ *hybridization that, on average, members of the SAR11 clade account for one-third of the ocean surface bacterioplankton communities [27].

#### Strategically located reference genomes

The use of metagenomics to phylotype communities has been limited by the lack of sequenced genomes from many taxonomic groups. To help fill some of these gaps, we sequenced representatives of several phyla for which genome sequences were not previously available. For example, we recently sequenced the genomes of *Dictyoglomus thermophilum *and *Thermomicrobium roseum *as part of a US National Science Foundation (NSF) funded 'Tree of life' project (Eisen JA and coworkers, unpublished data). To demonstrate the usefulness of these additional genomes for improved phylotyping, we analyzed metagenomic data from a Yellowstone hot spring community. From the 8,341 Sanger sequence reads obtained, we identified 59 reads that match the marker sequences present in our database. For 20 of these reads, their closest neighbors by phylogenetic analysis are *D. thermophilum *or *T. roseum *(ten reads each), thus demonstrating the usefulness of these genomes for phylotyping their close relatives in the Yellowstone community (see Additional data file 4 for one such example). This highlights the need to increase taxonomic sampling by selecting bacteria for sequencing based on their phylogenetic positions.

#### Selecting reference sequences

For best phylotyping results, the more reference sequences the better. Therefore, theoretically, the greater number of marker sequences identifiable from a more comprehensive database such as the NCBI nonredundant protein sequence (nr) database would be preferable to the lesser number obtainable from complete genomes. However, taxonomic sampling bias of the reference sequences has a great impact on the resulting phylotype assignments (see below). To be able to make meaningful comparisons among the results obtained using different markers, the taxonomic sampling must be controlled. In this regard, a complete genome database, in which every marker was sampled equally, would be preferable to the NCBI nr database, in which each marker was sampled to a different extent.

With very few exceptions such as gyrB [28], the protein marker sequences with species information in the nr database were mostly derived from genome sequencing projects. This is because it is very difficult to obtain protein encoding genes by polymerase chain reaction amplification because their sequences are not conserved at the nucleotide level [29]. As a result, the nr database does not actually contain many more protein marker sequences that can be used as references than those available from complete genome sequences.

#### Comparison of phylogeny-based and similarity-based phylotyping

Although our phylogeny-based phylotyping is fully automated, it still requires many more steps than, and is slower than, similarity based phylotyping methods such as a MEGAN [30]. Is it worth the trouble? Similarity based phylotyping works by searching a query sequence against a reference database such as NCBI nr and deriving taxonomic information from the best matches or 'hits'. When species that are closely related to the query sequence exist in the reference database, similarity-based phylotyping can work well. However, if the reference database is a biased sample or if it contains no closely related species to the query, then the top hits returned could be misleading [31]. Furthermore, similarity-based methods require an arbitrary similarity cut-off value to define the top hits. Because individual bacterial genomes and proteins can evolve at very different rates, a universal cut-off that works under all conditions does not exist. As a result, the final results can be very subjective.

In contrast, our tree-based bracketing algorithm places the query sequence within the context of a phylogenetic tree and only assigns it to a taxonomic level if that level has adequate sampling (see Materials and methods [below] for details of the algorithm). With the well sampled species *Prochlorococcus marinus*, for example, our method can distinguish closely related organisms and make taxonomic identifications at the species level. Our reanalysis of the Sargasso Sea data placed 672 sequences (3.6% of the total) within a *P. marinus *clade. On the other hand, for sparsely sampled clades such as *Aquifex*, assignments will be made only at the phylum level. Thus, our phylogeny-based analysis is less susceptible to data sampling bias than a similarity based approach, and it makes sequence assignments only at the taxonomic levels that are supported by the available data.

**Figure 4:**
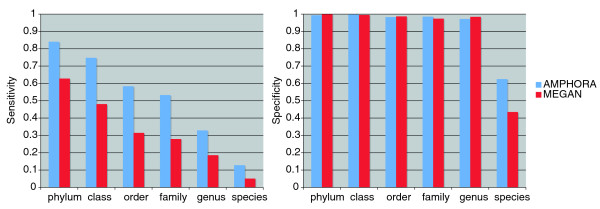
Comparison of the phylotyping performance by AMPHORA and MEGAN. The sensitivity and specificity of the phylotyping methods were measured across taxonomic ranks using simulated Sanger shotgun sequences of 31 genes from 100 representative bacterial genomes. The figure shows that AMPHORA significantly outperforms MEGAN in sensitivity without sacrificing specificity.

To compare quantitatively the performance of the phylogeny based and the similarity based phylotyping, we carried out a simulation study. We determined the sensitivity and specificity of the taxonomic assignments made by AMPHORA and MEGAN using 3,088 simulated shotgun sequences of 31 phylogenetic marker genes identified from 100 known bacterial genomes as benchmarks. The 100 genomes were chosen in such a way that maximizes their representation of the phylogenetic diversity and thus decreases the impact of the data sampling bias of current genome sequencing efforts on our results. Figure 4 compares the sensitivity and specificity of the phylotyping assignments at the phylum, class, order, family, and genus level using AMPHORA and MEGAN. The general trend toward decreasing sensitivity seen in the figure from the phylum to the species level simply reflects the fact that the amount of reference data available for taxonomic assignment is decreasing. However, AMPHORA significantly outperformed MEGAN in sensitivity at all taxonomic ranks. Both methods performed extremely well in specificity at all levels (>0.97) except at the species level, where AMPHORA (0.63) outperformed MEGAN (0.43) by a large margin.

### Future issues

#### Additional markers

We are in the process of adding more proteins to our initial database of 31 markers, including the commonly used protein markers RecA, HSP70, and EF-Tu. Ideally, a probability based method that evaluates the positional homology of the multiple sequence alignment could be developed to automate fully the process of masking. Major expansion will also require systematic assessment of many other protein families for their suitability as phylogenetic markers. For metagenomic phylotyping, the marker genes do not have to be single-copy or universal, but they must have been reasonably well sampled, have sufficient phylogenetic signal, and not be frequently exchanged between distantly related lineages. Until we learn more about the extent of lateral gene transfer in natural microbial communities, we caution against using every protein sequence collected in metagenomics studies for microbial diversity study.

#### More reference genomes

We have shown that adding representatives of novel phyla can facilitate metagenomic phylotyping. More reference genomes are needed for optimal performance. Although the sequencing of thousands of microbial genomes is underway, the organisms chosen are a biased sample and thus are not truly representative of the total microbial diversity. We see a need to select microbes systematically for sequencing based mainly on their phylogenetic positions, thus maximizing their value for comparative genomics and phylogenomic studies.

## Conclusion

Currently, SSU rRNA is still the most powerful phylogenetic marker because of the number of sequences available and the scope of taxonomic coverage. However, the imminent arrival of thousands of microbial genome sequences will vastly expand the amount of data available for alternative protein phylogenetic markers, thus presenting us with both a challenge and an opportunity. We have developed AMPHORA, a fully automated method for phylogenetic inference using multiple protein markers. AMPHORA offers speed, reliability, and high quality analyses. By eliminating the need for time consuming manual curation of sequence alignments, it removes one of the tightest bottlenecks in large-scale protein phylogenetic inference. We demonstrated its usefulness for automating both the construction of genome trees and the assignment of phylotypes to environmental metagenomic data. We believe such a phylogenomic approach will be valuable in helping us to make sense of rapidly accumulating microbial genomic data.

## Materials and methods

### Protein phylogenetic marker database

For each marker, we first identified their protein sequences from representative bacterial genomes. The amino acid sequences were aligned using CLUSTALW [32] and then manually edited and masked using the GDE package [33]. The mask is a text string of '1' and '0', where reliably aligned columns were labeled '1' and ambiguous columns were labeled '0'. Next, we used HMMer [34] to make local profile HMMs from these 'seed' alignments (Figure 1).

### Automated sequence alignment and trimming

Subsequent steps are carried out by a Perl script joining multiple automated processes (Figure 1). First, HMMer efficiently aligns the query amino acid sequences onto the trusted and fixed seed alignments. The Perl script then reads the masks embedded in the seed alignments and automatically trims the query alignments accordingly.

### Bacterial genome tree construction

Homologs of each of the 31 phylogenetic marker genes were identified from the 578 complete bacterial genomes by BLASTP searches (using marker sequences of *Escherichia coli *as query sequences and a cut-off E-value of 0.1) followed by HMMer searches (cut-off E-value 1 × e^-10^). The corresponding protein sequences were retrieved, aligned, and trimmed as described above, and then concatenated by species into a mega-alignment. A maximum likelihood tree was then constructed from the mega-alignment using PHYML [35]. The model selected based on the likelihood ratio test was the WAG model of amino acid substitution with γ-distributed rate variation (five categories) and a proportion of invariable sites. The shape of the γ-distribution and the proportion of the invariable sites were estimated by the program.

To speed up bootstrapping analyses, very closely related taxa were removed from the original mega-alignment, which left us with 310 taxa. Maximum likelihood trees were made from 100 bootstrapped replicates of this reduced dataset using PHYML with the same parameters described above.

With very few exceptions, the marker genes are single-copy genes in all of the bacterial genomes analyzed. In those rare cases in which two or more homologs were identified within a single species, a tree-guided approach was used to resolve the redundancy. If the redundancy resulted from a species-specific duplication event, then one homolog was randomly chosen as the representative. In all other cases, to avoid potential complications such as lateral gene transfer, we excluded that marker and treated it as 'missing' in that particular genome. It has been shown that as long as there is sufficient data, a few 'holes' in the dataset will not compromise the resulting tree [36].

### Phylotyping by phylogenetic analyses (AMPHORA)

The protein markers used to construct the bacterial genome tree (see above) and the resultant genome tree were used as the reference sequences and the reference tree for phylotyping metagenomic data from the Sargasso Sea or the simulated sequences described below. Each marker sequence identified from the metagenomic data or simulated sequences was individually aligned to its corresponding reference sequences and trimmed using the method described above. Then it was inserted into the reference tree using a maximum parsimony method of RAXML [37], constraining the topology of the tree to that of the genome tree. This tree construction procedure was extremely fast, and 100 bootstrap replicates were run for each query sequence to assess the confidence of the branching orders. The trees were rooted arbitrarily using *Deinococcus radiodurans *as the outgroup. Tree branch lengths were calculated using the neighbor joining algorithm with a fixed tree topology.

**Figure 5:**
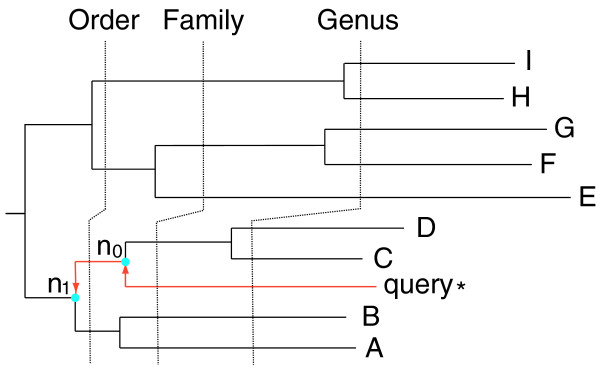
A tree based bracketing algorithm for phylotyping a query sequence. To assign a phylotype to the query sequence, its immediate ancestor *n*_0 _and the first internal node *n*_1 _with ≥70% bootstrapping support were identified. The known descendant leaf nodes of *n*_1_, namely A through D, are used to infer the taxonomy of the query, in conjunction with the normalized branch length information. The dashed timelines delimiting various taxonomic ranks were inferred from a clock that had been calibrated from the bacterial genome tree.

A tree-based bracketing algorithm was then employed to assign a phylotype to the query sequence (Figure 5), as follows. Starting from the immediate ancestor *n*_*0*_ of the query sequence and moving toward the root of the tree, the first internal node *n*_*1*_ whose bootstrap support exceeded a cut-off (for example, 70%) was identified. The common NCBI taxonomy *t*_*1*_ that was shared by all descendants of the node *n*_*1*_ represented the most conservative taxonomic prediction for the query sequence. Using the branch length information, finer scale phylotyping was carried out by comparing the normalized branch length from *n*_*0*_ to *n*_*1*_ with these between taxonomic ranks that had been tallied from the bacterial genome tree. Based on this comparison, a taxonomic rank below or equal to *t*_*1*_ was assigned to the node *n*_*0*_. The taxonomy of the sister node of the query at this rank was then assigned to the query. All tree branch lengths were normalized by dividing them by the lengths of the root-to-tip branches of their particular lineages. This was done to make the tree more clock-like, and therefore the branch lengths would be much more informative in inferring the time of evolution. In the simulation study, the query sequence itself was removed from the reference dataset before the analyses.

### Phylotyping by similarity-based analyses (MEGAN)

A total of 3,088 simulated phylogenetic marker gene sequences described below were searched against a database of complete bacterial genomes using BLASTX. The query sequence itself was discarded from the BLAST hits before feeding the BLAST results into the software MEGAN [30] for similarity-based phylotyping. A top per cent cut-off of 20% was used to retain only those hits whose matching scores are at least 80% of the best matching score. This cut-off was chosen to match the one used in a similar phylotyping simulation study described in the original MEGAN report [30]. All other parameters of MEGAN were set as default values except that the min-support (the minimum number of sequence reads that must be assigned to a taxon) is set to 1, because in our simulation study each query sequence was assigned a phylotype independently.

### Phylotyping simulation study

To assess the performance of the phylotyping methods, a simulation study was carried out. One hundred representative genomes maximizing the phylogenetic diversity of the 578 complete bacterial genomes were selected using the genome tree and an algorithm described in the report by Steel [38]. From each of the 31 phylogenetic marker genes identified from the 100 bacterial genomes, a DNA sequence fragment of 300 to 900 base pairs in length was randomly chosen, which resulted in a total of 3,088 simulated shotgun sequences that were used as benchmark query sequences in phylotyping (some markers are missing in some of the genomes). By comparing the predicted taxa with the known taxa, the sensitivity and specificity of phylotyping methods were calculated as described in the report by Krause and coworkers [39]. Briefly, for a taxon *i*, let *P*_*i *_be the number of query sequences from *i*, *TP*_*i *_be the number of sequences that are correctly assigned to *i*, and *FP*_*i *_be the number of sequences that are incorrectly assigned to *i*. The sensitivity *TP*_*i*_/*P*_*i *_measures the proportion of query sequences that are correctly classified. The specificity *TP*_*i*_/(*TP*_*i *_+ *FP*_*i*_) measures the reliability of the phylotyping assignments.

### SSU rRNA tree construction

SSU rDNA sequences were extracted from complete genomes, aligned, and trimmed using an online tool MyRDP [40]. When multiple copies of SSU rRNA genes were present within a single genome, one representative was randomly chosen. Maximum likelihood tree was constructed using PHYML [35], applying the GTR model of substitution, with a γ-distribution (α estimated by the program) of rates of five categories of variable sites and a proportion of invariable sites (proportion estimated by the program).

## Availability

The AMPHORA package and the simulation study data can be downloaded from [41].

## Abbreviations

AMPHORA: AutoMated PHylogenOmic infeRence; HMM: hidden Markov model; NCBI: National Center for Biotechnology Information; nr: nonredundant protein sequence; SSU: small subunit NSF: US National Science Foundation.

## Authors' contributions

MW designed the study, developed the method, and performed the analyses. JAE advised on method design and testing. MW and JAE wrote the paper.

## Additional data files

The following additional data are available with the online version of this paper. Additional data file 1 is a table listing the 578 complete bacterial genomes downloaded from the NCBI RefSeq database for this study. Additional data file 2 is a figure of a maximum likelihood genome tree of 578 bacterial species; major taxonomic groups are highlighted by color. Additional data file 3 provides a figure that compares γ-proteobacterial phylogenetic trees made from a super-matrix of 31 protein phylogenetic markers and from the SSU rDNA; bootstrap support values are shown along their corresponding branches. Additional data file 4 is a figure of a maximum likelihood tree of rpoB; adding a novel genome (*Thermomicrobium roseum*) to the reference tree helped anchor a sequence read (ZAVAM73TF) from a Yellowstone hotspring metagenomic study. Additional data file 5 is a table listing phylotypes breakdown of the Sargasso Sea metagenomic sequence data by phylogenetic markers and major taxonomic groups.

## Supplementary Material

Additional data file 1 Presented is a table listing the 578 complete bacterial genomes downloaded from the NCBI RefSeq database for this study. Click here for file

Additional data file 2 Presented is a figure of a maximum likelihood genome tree of 578 bacterial species. Major taxonomic groups are highlighted by color. Click here for file

Additional data file 3 Presented is a figure that compares γ-proteobacterial phylogenetic trees made from a super-matrix of 31 protein phylogenetic markers (A) and from the SSU rDNA (B). Bootstrap support values are shown along their corresponding branches. Click here for file

Additional data file 4 Presented is a figure of a maximum likelihood tree of rpoB. Adding a novel genome (*Thermomicrobium roseum*) to the reference tree helped anchor a sequence read (ZAVAM73TF) from a Yellowstone hotspring metagenomic study. Click here for file

Additional data file 5 Presented is a table listing phylotypes breakdown of the Sargasso Sea metagenomic sequence data by phylogenetic markers and major taxonomic groups. Click here for file
