# Supplementary material for: A simple, fast, and accurate method of phylogenomic inference
Source: Genome Biol. 2008 Oct 13;9(10):R151. doi: 10.1186/gb-2008-9-10-r151 (PMC2760878; doi:10.1186/gb-2008-9-10-r151)
Supplement: Additional data file 1 — Presented is a table listing the 578 complete bacterial genomes downloaded from the NCBI RefSeq database for this study. [file gb-2008-9-10-r151-S1.doc]

| **Complete bacterial genomes** | **Chromosome RefSeq Accession #** |
| --- | --- |
| Acaryochloris marina MBIC11017 | NC_009925 |
| Acholeplasma laidlawii PG-8A | NC_010163 |
| Acidiphilium cryptum JF-5 | NC_009484 |
| Acidobacteria bacterium Ellin345 | NC_008009 |
| Acidothermus cellulolyticus 11B | NC_008578 |
| Acidovorax avenae subsp. citrulli AAC00-1 | NC_008752 |
| Acidovorax sp. JS42 | NC_008782 |
| Acinetobacter baumannii ATCC 17978 | NC_009085 |
| Acinetobacter sp. ADP1 | NC_005966 |
| Actinobacillus pleuropneumoniae L20 | NC_009053 |
| Actinobacillus pleuropneumoniae serovar 3 str. JL03 | NC_010278 |
| Actinobacillus succinogenes 130Z | NC_009655 |
| Aeromonas hydrophila subsp. hydrophila ATCC 7966 | NC_008570 |
| Aeromonas salmonicida subsp. salmonicida A449 | NC_009348 |
| Agrobacterium tumefaciens str. C58 | NC_003305 NC_003304 |
| Alcanivorax borkumensis SK2 | NC_008260 |
| Alkalilimnicola ehrlichei MLHE-1 | NC_008340 |
| Alkaliphilus metalliredigens QYMF | NC_009633 |
| Alkaliphilus oremlandii OhILAs | NC_009922 |
| Anabaena variabilis ATCC 29413 | NC_007413 |
| Anaeromyxobacter dehalogenans 2CP-C | NC_007760 |
| Anaeromyxobacter sp. Fw109-5 | NC_009675 |
| Anaplasma marginale str. St. Maries | NC_004842 |
| Anaplasma phagocytophilum HZ | NC_007797 |
| Aquifex aeolicus VF5 | NC_000918 |
| Arcobacter butzleri RM4018 | NC_009850 |
| Arthrobacter aurescens TC1 | NC_008711 |
| Arthrobacter sp. FB24 | NC_008541 |
| Aster yellows witches'-broom phytoplasma AYWB | NC_007716 |
| Azoarcus sp. BH72 | NC_008702 |
| Azoarcus sp. EbN1 | NC_006513 |
| Azorhizobium caulinodans ORS 571 | NC_009937 |
| Bacillus amyloliquefaciens FZB42 | NC_009725 |
| Bacillus anthracis str. 'Ames Ancestor' | NC_007530 |
| Bacillus anthracis str. Ames | NC_003997 |
| Bacillus anthracis str. Sterne | NC_005945 |
| Bacillus cereus ATCC 10987 | NC_003909 |
| Bacillus cereus ATCC 14579 | NC_004722 |
| Bacillus cereus E33L | NC_006274 |
| Bacillus cereus subsp. cytotoxis NVH 391-98 | NC_009674 |
| Bacillus clausii KSM-K16 | NC_006582 |
| Bacillus halodurans C-125 | NC_002570 |
| Bacillus licheniformis ATCC 14580 | NC_006270 |
| Bacillus pumilus SAFR-032 | NC_009848 |
| Bacillus subtilis subsp. subtilis str. 168 | NC_000964 |
| Bacillus thuringiensis serovar konkukian str. 97-27 | NC_005957 |
| Bacillus thuringiensis str. Al Hakam | NC_008600 |
| Bacillus weihenstephanensis KBAB4 | NC_010184 |
| Bacteroides fragilis NCTC 9343 | NC_003228 |
| Bacteroides fragilis YCH46 | NC_006347 |
| Bacteroides thetaiotaomicron VPI-5482 | NC_004663 |
| Bacteroides vulgatus ATCC 8482 | NC_009614 |
| Bartonella bacilliformis KC583 | NC_008783 |
| Bartonella henselae str. Houston-1 | NC_005956 |
| Bartonella quintana str. Toulouse | NC_005955 |
| Bartonella tribocorum CIP 105476 | NC_010161 |
| Baumannia cicadellinicola str. Hc (Homalodisca coagulata) | NC_007984 |
| Bdellovibrio bacteriovorus HD100 | NC_005363 |
| Bifidobacterium adolescentis ATCC 15703 | NC_008618 |
| Bifidobacterium longum NCC2705 | NC_004307 |
| Bordetella bronchiseptica RB50 | NC_002927 |
| Bordetella parapertussis 12822 | NC_002928 |
| Bordetella pertussis Tohama I | NC_002929 |
| Bordetella petrii | NC_010170 |
| Borrelia afzelii PKo | NC_008277 |
| Borrelia burgdorferi B31 | NC_001318 |
| Borrelia garinii PBi | NC_006156 |
| Bradyrhizobium japonicum USDA 110 | NC_004463 |
| Bradyrhizobium sp. BTAi1 | NC_009485 |
| Bradyrhizobium sp. ORS278 | NC_009445 |
| Brucella abortus biovar 1 str. 9-941 | NC_006933 NC_006932 |
| Brucella canis ATCC 23365 | NC_010104 NC_010103 |
| Brucella melitensis 16M | NC_003317 NC_003318 |
| Brucella melitensis biovar Abortus 2308 | NC_007618 NC_007624 |
| Brucella ovis ATCC 25840 | NC_009504 NC_009505 |
| Brucella suis 1330 | NC_004310 NC_004311 |
| Brucella suis ATCC 23445 | NC_010169 NC_010167 |
| Buchnera aphidicola str. APS (Acyrthosiphon pisum) | NC_002528 |
| Buchnera aphidicola str. Bp (Baizongia pistaciae) | NC_004545 |
| Buchnera aphidicola str. Cc (Cinara cedri) | NC_008513 |
| Buchnera aphidicola str. Sg (Schizaphis graminum) | NC_004061 |
| Burkholderia ambifaria AMMD | NC_008390 NC_008392 NC_008391 |
| Burkholderia cenocepacia AU 1054 | NC_008061 NC_008060 NC_008062 |
| Burkholderia cenocepacia HI2424 | NC_008543 NC_008542 NC_008544 |
| Burkholderia mallei ATCC 23344 | NC_006348 NC_006349 |
| Burkholderia mallei NCTC 10229 | NC_008835 NC_008836 |
| Burkholderia mallei NCTC 10247 | NC_009079 NC_009080 |
| Burkholderia mallei SAVP1 | NC_008785 NC_008784 |
| Burkholderia multivorans ATCC 17616 | NC_010087 NC_010086 NC_010084 |
| Burkholderia pseudomallei 1106a | NC_009076 NC_009078 |
| Burkholderia pseudomallei 1710b | NC_007434 NC_007435 |
| Burkholderia pseudomallei 668 | NC_009074 NC_009075 |
| Burkholderia pseudomallei K96243 | NC_006351 NC_006350 |
| Burkholderia sp. 383 | NC_007511 NC_007510 NC_007509 |
| Burkholderia thailandensis E264 | NC_007651 NC_007650 |
| Burkholderia vietnamiensis G4 | NC_009256 NC_009254 NC_009255 |
| Burkholderia xenovorans LB400 | NC_007952 NC_007953 NC_007951 |
| Caldicellulosiruptor saccharolyticus DSM 8903 | NC_009437 |
| Campylobacter concisus 13826 | NC_009802 |
| Campylobacter curvus 525.92 | NC_009715 |
| Campylobacter fetus subsp. fetus 82-40 | NC_008599 |
| Campylobacter hominis ATCC BAA-381 | NC_009714 |
| Campylobacter jejuni RM1221 | NC_003912 |
| Campylobacter jejuni subsp. doylei 269.97 | NC_009707 |
| Campylobacter jejuni subsp. jejuni 81-176 | NC_008787 |
| Campylobacter jejuni subsp. jejuni 81116 | NC_009839 |
| Campylobacter jejuni subsp. jejuni NCTC 11168 | NC_002163 |
| Candidatus Blochmannia floridanus | NC_005061 |
| Candidatus Blochmannia pennsylvanicus str. BPEN | NC_007292 |
| Candidatus Pelagibacter ubique HTCC1062 | NC_007205 |
| Candidatus Protochlamydia amoebophila UWE25 | NC_005861 |
| Candidatus Ruthia magnifica str. Cm (Calyptogena magnifica) | NC_008610 |
| Candidatus Sulcia muelleri GWSS | NC_010118 |
| Candidatus Vesicomyosocius okutanii HA | NC_009465 |
| Carboxydothermus hydrogenoformans Z-2901 | NC_007503 |
| Caulobacter crescentus CB15 | NC_002696 |
| Chlamydia muridarum Nigg | NC_002620 |
| Chlamydia trachomatis 434/Bu | NC_010287 |
| Chlamydia trachomatis A/HAR-13 | NC_007429 |
| Chlamydia trachomatis D/UW-3/CX | NC_000117 |
| Chlamydia trachomatis L2b/UCH-1/proctitis | NC_010280 |
| Chlamydophila abortus S26/3 | NC_004552 |
| Chlamydophila caviae GPIC | NC_003361 |
| Chlamydophila felis Fe/C-56 | NC_007899 |
| Chlamydophila pneumoniae AR39 | NC_002179 |
| Chlamydophila pneumoniae CWL029 | NC_000922 |
| Chlamydophila pneumoniae J138 | NC_002491 |
| Chlamydophila pneumoniae TW-183 | NC_005043 |
| Chlorobium chlorochromatii CaD3 | NC_007514 |
| Chlorobium phaeobacteroides DSM 266 | NC_008639 |
| Chlorobium tepidum TLS | NC_002932 |
| Chloroflexus aurantiacus J-10-fl | NC_010175 |
| Chromobacterium violaceum ATCC 12472 | NC_005085 |
| Chromohalobacter salexigens DSM 3043 | NC_007963 |
| Citrobacter koseri ATCC BAA-895 | NC_009792 |
| Clavibacter michiganensis subsp. michiganensis NCPPB 382 | NC_009480 |
| Clostridium acetobutylicum ATCC 824 | NC_003030 |
| Clostridium beijerinckii NCIMB 8052 | NC_009617 |
| Clostridium botulinum A str. ATCC 19397 | NC_009697 |
| Clostridium botulinum A str. ATCC 3502 | NC_009495 |
| Clostridium botulinum A str. Hall | NC_009698 |
| Clostridium botulinum F str. Langeland | NC_009699 |
| Clostridium difficile 630 | NC_009089 |
| Clostridium kluyveri DSM 555 | NC_009706 |
| Clostridium novyi NT | NC_008593 |
| Clostridium perfringens ATCC 13124 | NC_008261 |
| Clostridium perfringens SM101 | NC_008262 |
| Clostridium perfringens str. 13 | NC_003366 |
| Clostridium phytofermentans ISDg | NC_010001 |
| Clostridium tetani E88 | NC_004557 |
| Clostridium thermocellum ATCC 27405 | NC_009012 |
| Colwellia psychrerythraea 34H | NC_003910 |
| Corynebacterium diphtheriae NCTC 13129 | NC_002935 |
| Corynebacterium efficiens YS-314 | NC_004369 |
| Corynebacterium glutamicum ATCC 13032 | NC_003450 |
| Corynebacterium glutamicum R | NC_009342 |
| Corynebacterium jeikeium K411 | NC_007164 |
| Coxiella burnetii Dugway 5J108-111 | NC_009727 |
| Coxiella burnetii RSA 331 | NC_010117 |
| Coxiella burnetii RSA 493 | NC_002971 |
| Cytophaga hutchinsonii ATCC 33406 | NC_008255 |
| Dechloromonas aromatica RCB | NC_007298 |
| Dehalococcoides ethenogenes 195 | NC_002936 |
| Dehalococcoides sp. BAV1 | NC_009455 |
| Dehalococcoides sp. CBDB1 | NC_007356 |
| Deinococcus geothermalis DSM 11300 | NC_008025 |
| Deinococcus radiodurans R1 | NC_001263 NC_001264 |
| Delftia acidovorans SPH-1 | NC_010002 |
| Desulfitobacterium hafniense Y51 | NC_007907 |
| Desulfococcus oleovorans Hxd3 | NC_009943 |
| Desulfotalea psychrophila LSv54 | NC_006138 |
| Desulfotomaculum reducens MI-1 | NC_009253 |
| Desulfovibrio desulfuricans G20 | NC_007519 |
| Desulfovibrio vulgaris subsp. vulgaris DP4 | NC_008751 |
| Desulfovibrio vulgaris subsp. vulgaris str. Hildenborough | NC_002937 |
| Dichelobacter nodosus VCS1703A | NC_009446 |
| Dinoroseobacter shibae DFL 12 | NC_009952 |
| Ehrlichia canis str. Jake | NC_007354 |
| Ehrlichia chaffeensis str. Arkansas | NC_007799 |
| Ehrlichia ruminantium str. Gardel | NC_006831 |
| Ehrlichia ruminantium str. Welgevonden | NC_005295 |
| Enterobacter sakazakii ATCC BAA-894 | NC_009778 |
| Enterobacter sp. 638 | NC_009436 |
| Enterococcus faecalis V583 | NC_004668 |
| Erwinia carotovora subsp. atroseptica SCRI1043 | NC_004547 |
| Erythrobacter litoralis HTCC2594 | NC_007722 |
| Escherichia coli 536 | NC_008253 |
| Escherichia coli APEC O1 | NC_008563 |
| Escherichia coli CFT073 | NC_004431 |
| Escherichia coli E24377A | NC_009801 |
| Escherichia coli HS | NC_009800 |
| Escherichia coli K12 | NC_000913 |
| Escherichia coli O157:H7 EDL933 | NC_002655 |
| Escherichia coli O157:H7 str. Sakai | NC_002695 |
| Escherichia coli UTI89 | NC_007946 |
| Fervidobacterium nodosum Rt17-B1 | NC_009718 |
| Flavobacterium johnsoniae UW101 | NC_009441 |
| Flavobacterium psychrophilum JIP02/86 | NC_009613 |
| Francisella tularensis subsp. holarctica | NC_007880 |
| Francisella tularensis subsp. holarctica FTA | NC_009749 |
| Francisella tularensis subsp. holarctica OSU18 | NC_008369 |
| Francisella tularensis subsp. novicida U112 | NC_008601 |
| Francisella tularensis subsp. tularensis FSC198 | NC_008245 |
| Francisella tularensis subsp. tularensis SCHU S4 | NC_006570 |
| Francisella tularensis subsp. tularensis WY96-3418 | NC_009257 |
| Frankia alni ACN14a | NC_008278 |
| Frankia sp. CcI3 | NC_007777 |
| Frankia sp. EAN1pec | NC_009921 |
| Fusobacterium nucleatum subsp. nucleatum ATCC 25586 | NC_003454 |
| Geobacillus kaustophilus HTA426 | NC_006510 |
| Geobacillus thermodenitrificans NG80-2 | NC_009328 |
| Geobacter metallireducens GS-15 | NC_007517 |
| Geobacter sulfurreducens PCA | NC_002939 |
| Geobacter uraniireducens Rf4 | NC_009483 |
| Gloeobacter violaceus PCC 7421 | NC_005125 |
| Gluconacetobacter diazotrophicus PAl 5 | NC_010125 |
| Gluconobacter oxydans 621H | NC_006677 |
| Gramella forsetii KT0803 | NC_008571 |
| Granulibacter bethesdensis CGDNIH1 | NC_008343 |
| Haemophilus ducreyi 35000HP | NC_002940 |
| Haemophilus influenzae 86-028NP | NC_007146 |
| Haemophilus influenzae PittEE | NC_009566 |
| Haemophilus influenzae PittGG | NC_009567 |
| Haemophilus influenzae Rd KW20 | NC_000907 |
| Haemophilus somnus 129PT | NC_008309 |
| Hahella chejuensis KCTC 2396 | NC_007645 |
| Halorhodospira halophila SL1 | NC_008789 |
| Helicobacter acinonychis str. Sheeba | NC_008229 |
| Helicobacter hepaticus ATCC 51449 | NC_004917 |
| Helicobacter pylori 26695 | NC_000915 |
| Helicobacter pylori HPAG1 | NC_008086 |
| Helicobacter pylori J99 | NC_000921 |
| Herminiimonas arsenicoxydans | NC_009138 |
| Herpetosiphon aurantiacus ATCC 23779 | NC_009972 |
| Hyphomonas neptunium ATCC 15444 | NC_008358 |
| Idiomarina loihiensis L2TR | NC_006512 |
| Jannaschia sp. CCS1 | NC_007802 |
| Janthinobacterium sp. Marseille | NC_009659 |
| Kineococcus radiotolerans SRS30216 | NC_009664 |
| Klebsiella pneumoniae subsp. pneumoniae MGH 78578 | NC_009648 |
| Lactobacillus acidophilus NCFM | NC_006814 |
| Lactobacillus brevis ATCC 367 | NC_008497 |
| Lactobacillus casei ATCC 334 | NC_008526 |
| Lactobacillus delbrueckii subsp. bulgaricus ATCC 11842 | NC_008054 |
| Lactobacillus delbrueckii subsp. bulgaricus ATCC BAA-365 | NC_008529 |
| Lactobacillus gasseri ATCC 33323 | NC_008530 |
| Lactobacillus helveticus DPC 4571 | NC_010080 |
| Lactobacillus johnsonii NCC 533 | NC_005362 |
| Lactobacillus plantarum WCFS1 | NC_004567 |
| Lactobacillus reuteri F275 | NC_009513 |
| Lactobacillus sakei subsp. sakei 23K | NC_007576 |
| Lactobacillus salivarius UCC118 | NC_007929 |
| Lactococcus lactis subsp. cremoris MG1363 | NC_009004 |
| Lactococcus lactis subsp. cremoris SK11 | NC_008527 |
| Lactococcus lactis subsp. lactis Il1403 | NC_002662 |
| Lawsonia intracellularis PHE/MN1-00 | NC_008011 |
| Legionella pneumophila str. Corby | NC_009494 |
| Legionella pneumophila str. Lens | NC_006369 |
| Legionella pneumophila str. Paris | NC_006368 |
| Legionella pneumophila subsp. pneumophila str. Philadelphia 1 | NC_002942 |
| Leifsonia xyli subsp. xyli str. CTCB07 | NC_006087 |
| Leptospira borgpetersenii serovar Hardjo-bovis JB197 | NC_008510 NC_008511 |
| Leptospira borgpetersenii serovar Hardjo-bovis L550 | NC_008508 NC_008509 |
| Leptospira interrogans serovar Copenhageni str. Fiocruz L1-130 | NC_005823 NC_005824 |
| Leptospira interrogans serovar Lai str. 56601 | NC_004342 NC_004343 |
| Leuconostoc mesenteroides subsp. mesenteroides ATCC 8293 | NC_008531 |
| Listeria innocua Clip11262 | NC_003212 |
| Listeria monocytogenes EGD-e | NC_003210 |
| Listeria monocytogenes str. 4b F2365 | NC_002973 |
| Listeria welshimeri serovar 6b str. SLCC5334 | NC_008555 |
| Magnetococcus sp. MC-1 | NC_008576 |
| Magnetospirillum magneticum AMB-1 | NC_007626 |
| Mannheimia succiniciproducens MBEL55E | NC_006300 |
| Maricaulis maris MCS10 | NC_008347 |
| Marinobacter aquaeolei VT8 | NC_008740 |
| Marinomonas sp. MWYL1 | NC_009654 |
| Mesoplasma florum L1 | NC_006055 |
| Mesorhizobium loti MAFF303099 | NC_002678 |
| Mesorhizobium sp. BNC1 | NC_008254 |
| Methylibium petroleiphilum PM1 | NC_008825 |
| Methylobacillus flagellatus KT | NC_007947 |
| Methylobacterium extorquens PA1 | NC_010172 |
| Methylococcus capsulatus str. Bath | NC_002977 |
| Microcystis aeruginosa NIES-843 | NC_010296 |
| Moorella thermoacetica ATCC 39073 | NC_007644 |
| Mycobacterium avium 104 | NC_008595 |
| Mycobacterium avium subsp. paratuberculosis K-10 | NC_002944 |
| Mycobacterium bovis AF2122/97 | NC_002945 |
| Mycobacterium bovis BCG str. Pasteur 1173P2 | NC_008769 |
| Mycobacterium gilvum PYR-GCK | NC_009338 |
| Mycobacterium leprae TN | NC_002677 |
| Mycobacterium smegmatis str. MC2 155 | NC_008596 |
| Mycobacterium sp. JLS | NC_009077 |
| Mycobacterium sp. KMS | NC_008705 |
| Mycobacterium sp. MCS | NC_008146 |
| Mycobacterium tuberculosis CDC1551 | NC_002755 |
| Mycobacterium tuberculosis F11 | NC_009565 |
| Mycobacterium tuberculosis H37Ra | NC_009525 |
| Mycobacterium tuberculosis H37Rv | NC_000962 |
| Mycobacterium ulcerans Agy99 | NC_008611 |
| Mycobacterium vanbaalenii PYR-1 | NC_008726 |
| Mycoplasma agalactiae PG2 | NC_009497 |
| Mycoplasma capricolum subsp. capricolum ATCC 27343 | NC_007633 |
| Mycoplasma gallisepticum R | NC_004829 |
| Mycoplasma genitalium G37 | NC_000908 |
| Mycoplasma hyopneumoniae 232 | NC_006360 |
| Mycoplasma hyopneumoniae 7448 | NC_007332 |
| Mycoplasma hyopneumoniae J | NC_007295 |
| Mycoplasma mobile 163K | NC_006908 |
| Mycoplasma mycoides subsp. mycoides SC str. PG1 | NC_005364 |
| Mycoplasma penetrans HF-2 | NC_004432 |
| Mycoplasma pneumoniae M129 | NC_000912 |
| Mycoplasma pulmonis UAB CTIP | NC_002771 |
| Mycoplasma synoviae 53 | NC_007294 |
| Myxococcus xanthus DK 1622 | NC_008095 |
| Neisseria gonorrhoeae FA 1090 | NC_002946 |
| Neisseria meningitidis 053442 | NC_010120 |
| Neisseria meningitidis FAM18 | NC_008767 |
| Neisseria meningitidis MC58 | NC_003112 |
| Neisseria meningitidis Z2491 | NC_003116 |
| Neorickettsia sennetsu str. Miyayama | NC_007798 |
| Nitratiruptor sp. SB155-2 | NC_009662 |
| Nitrobacter hamburgensis X14 | NC_007964 |
| Nitrobacter winogradskyi Nb-255 | NC_007406 |
| Nitrosococcus oceani ATCC 19707 | NC_007484 |
| Nitrosomonas europaea ATCC 19718 | NC_004757 |
| Nitrosomonas eutropha C91 | NC_008344 |
| Nitrosospira multiformis ATCC 25196 | NC_007614 |
| Nocardia farcinica IFM 10152 | NC_006361 |
| Nocardioides sp. JS614 | NC_008699 |
| Nostoc sp. PCC 7120 | NC_003272 |
| Novosphingobium aromaticivorans DSM 12444 | NC_007794 |
| Oceanobacillus iheyensis HTE831 | NC_004193 |
| Ochrobactrum anthropi ATCC 49188 | NC_009667 NC_009668 |
| Oenococcus oeni PSU-1 | NC_008528 |
| Onion yellows phytoplasma OY-M | NC_005303 |
| Orientia tsutsugamushi Boryong | NC_009488 |
| Parabacteroides distasonis ATCC 8503 | NC_009615 |
| Paracoccus denitrificans PD1222 | NC_008686 NC_008687 |
| Parvibaculum lavamentivorans DS-1 | NC_009719 |
| Pasteurella multocida subsp. multocida str. Pm70 | NC_002663 |
| Pediococcus pentosaceus ATCC 25745 | NC_008525 |
| Pelobacter carbinolicus DSM 2380 | NC_007498 |
| Pelobacter propionicus DSM 2379 | NC_008609 |
| Pelodictyon luteolum DSM 273 | NC_007512 |
| Pelotomaculum thermopropionicum SI | NC_009454 |
| Petrotoga mobilis SJ95 | NC_010003 |
| Photobacterium profundum SS9 | NC_006371 NC_006370 |
| Photorhabdus luminescens subsp. laumondii TTO1 | NC_005126 |
| Polaromonas naphthalenivorans CJ2 | NC_008781 |
| Polaromonas sp. JS666 | NC_007948 |
| Polynucleobacter sp. QLW-P1DMWA-1 | NC_009379 |
| Porphyromonas gingivalis W83 | NC_002950 |
| Prochlorococcus marinus str. AS9601 | NC_008816 |
| Prochlorococcus marinus str. MIT 9211 | NC_009976 |
| Prochlorococcus marinus str. MIT 9215 | NC_009840 |
| Prochlorococcus marinus str. MIT 9301 | NC_009091 |
| Prochlorococcus marinus str. MIT 9303 | NC_008820 |
| Prochlorococcus marinus str. MIT 9312 | NC_007577 |
| Prochlorococcus marinus str. MIT 9313 | NC_005071 |
| Prochlorococcus marinus str. MIT 9515 | NC_008817 |
| Prochlorococcus marinus str. NATL1A | NC_008819 |
| Prochlorococcus marinus str. NATL2A | NC_007335 |
| Prochlorococcus marinus subsp. marinus str. CCMP1375 | NC_005042 |
| Prochlorococcus marinus subsp. pastoris str. CCMP1986 | NC_005072 |
| Propionibacterium acnes KPA171202 | NC_006085 |
| Prosthecochloris vibrioformis DSM 265 | NC_009337 |
| Pseudoalteromonas atlantica T6c | NC_008228 |
| Pseudoalteromonas haloplanktis TAC125 | NC_007482 NC_007481 |
| Pseudomonas aeruginosa PA7 | NC_009656 |
| Pseudomonas aeruginosa PAO1 | NC_002516 |
| Pseudomonas aeruginosa UCBPP-PA14 | NC_008463 |
| Pseudomonas entomophila L48 | NC_008027 |
| Pseudomonas fluorescens Pf-5 | NC_004129 |
| Pseudomonas fluorescens PfO-1 | NC_007492 |
| Pseudomonas mendocina ymp | NC_009439 |
| Pseudomonas putida F1 | NC_009512 |
| Pseudomonas putida GB-1 | NC_010322 |
| Pseudomonas putida KT2440 | NC_002947 |
| Pseudomonas stutzeri A1501 | NC_009434 |
| Pseudomonas syringae pv. phaseolicola 1448A | NC_005773 |
| Pseudomonas syringae pv. syringae B728a | NC_007005 |
| Pseudomonas syringae pv. tomato str. DC3000 | NC_004578 |
| Psychrobacter arcticus 273-4 | NC_007204 |
| Psychrobacter cryohalolentis K5 | NC_007969 |
| Psychrobacter sp. PRwf-1 | NC_009524 |
| Psychromonas ingrahamii 37 | NC_008709 |
| Ralstonia eutropha H16 | NC_008313 NC_008314 |
| Ralstonia eutropha JMP134 | NC_007348 NC_007347 |
| Ralstonia metallidurans CH34 | NC_007973 NC_007974 |
| Ralstonia solanacearum GMI1000 | NC_003295 |
| Renibacterium salmoninarum ATCC 33209 | NC_010168 |
| Rhizobium etli CFN 42 | NC_007761 |
| Rhizobium leguminosarum bv. viciae 3841 | NC_008380 |
| Rhodobacter sphaeroides 2.4.1 | NC_007494 NC_007493 |
| Rhodobacter sphaeroides ATCC 17025 | NC_009428 |
| Rhodobacter sphaeroides ATCC 17029 | NC_009049 NC_009050 |
| Rhodococcus sp. RHA1 | NC_008268 |
| Rhodoferax ferrireducens T118 | NC_007908 |
| Rhodopirellula baltica SH 1 | NC_005027 |
| Rhodopseudomonas palustris BisA53 | NC_008435 |
| Rhodopseudomonas palustris BisB18 | NC_007925 |
| Rhodopseudomonas palustris BisB5 | NC_007958 |
| Rhodopseudomonas palustris CGA009 | NC_005296 |
| Rhodopseudomonas palustris HaA2 | NC_007778 |
| Rhodospirillum rubrum ATCC 11170 | NC_007643 |
| Rickettsia akari str. Hartford | NC_009881 |
| Rickettsia bellii OSU 85-389 | NC_009883 |
| Rickettsia bellii RML369-C | NC_007940 |
| Rickettsia canadensis str. McKiel | NC_009879 |
| Rickettsia conorii str. Malish 7 | NC_003103 |
| Rickettsia felis URRWXCal2 | NC_007109 |
| Rickettsia massiliae MTU5 | NC_009900 |
| Rickettsia prowazekii str. Madrid E | NC_000963 |
| Rickettsia rickettsii str. 'Sheila Smith' | NC_009882 |
| Rickettsia rickettsii str. Iowa | NC_010263 |
| Rickettsia typhi str. Wilmington | NC_006142 |
| Roseiflexus castenholzii DSM 13941 | NC_009767 |
| Roseiflexus sp. RS-1 | NC_009523 |
| Roseobacter denitrificans OCh 114 | NC_008209 |
| Rubrobacter xylanophilus DSM 9941 | NC_008148 |
| Saccharophagus degradans 2-40 | NC_007912 |
| Saccharopolyspora erythraea NRRL 2338 | NC_009142 |
| Salinibacter ruber DSM 13855 | NC_007677 |
| Salinispora arenicola CNS-205 | NC_009953 |
| Salinispora tropica CNB-440 | NC_009380 |
| Salmonella enterica subsp. arizonae serovar 62:z4,z23:-- | NC_010067 |
| Salmonella enterica subsp. enterica serovar Choleraesuis str. SC-B67 | NC_006905 |
| Salmonella enterica subsp. enterica serovar Paratyphi A str. ATCC 9150 | NC_006511 |
| Salmonella enterica subsp. enterica serovar Paratyphi B str. SPB7 | NC_010102 |
| Salmonella enterica subsp. enterica serovar Typhi str. CT18 | NC_003198 |
| Salmonella enterica subsp. enterica serovar Typhi str. Ty2 | NC_004631 |
| Salmonella typhimurium LT2 | NC_003197 |
| Serratia proteamaculans 568 | NC_009832 |
| Shewanella amazonensis SB2B | NC_008700 |
| Shewanella baltica OS155 | NC_009052 |
| Shewanella baltica OS185 | NC_009665 |
| Shewanella baltica OS195 | NC_009997 |
| Shewanella denitrificans OS217 | NC_007954 |
| Shewanella frigidimarina NCIMB 400 | NC_008345 |
| Shewanella loihica PV-4 | NC_009092 |
| Shewanella oneidensis MR-1 | NC_004347 |
| Shewanella pealeana ATCC 700345 | NC_009901 |
| Shewanella putrefaciens CN-32 | NC_009438 |
| Shewanella sediminis HAW-EB3 | NC_009831 |
| Shewanella sp. ANA-3 | NC_008577 |
| Shewanella sp. MR-4 | NC_008321 |
| Shewanella sp. MR-7 | NC_008322 |
| Shewanella sp. W3-18-1 | NC_008750 |
| Shigella boydii Sb227 | NC_007613 |
| Shigella dysenteriae Sd197 | NC_007606 |
| Shigella flexneri 2a str. 2457T | NC_004741 |
| Shigella flexneri 2a str. 301 | NC_004337 |
| Shigella flexneri 5 str. 8401 | NC_008258 |
| Shigella sonnei Ss046 | NC_007384 |
| Silicibacter pomeroyi DSS-3 | NC_003911 |
| Silicibacter sp. TM1040 | NC_008044 |
| Sinorhizobium medicae WSM419 | NC_009636 |
| Sinorhizobium meliloti 1021 | NC_003047 |
| Sodalis glossinidius str. 'morsitans' | NC_007712 |
| Solibacter usitatus Ellin6076 | NC_008536 |
| Sorangium cellulosum 'So ce 56' | NC_010162 |
| Sphingomonas wittichii RW1 | NC_009511 |
| Sphingopyxis alaskensis RB2256 | NC_008048 |
| Staphylococcus aureus RF122 | NC_007622 |
| Staphylococcus aureus subsp. aureus COL | NC_002951 |
| Staphylococcus aureus subsp. aureus JH1 | NC_009632 |
| Staphylococcus aureus subsp. aureus JH9 | NC_009487 |
| Staphylococcus aureus subsp. aureus MRSA252 | NC_002952 |
| Staphylococcus aureus subsp. aureus MSSA476 | NC_002953 |
| Staphylococcus aureus subsp. aureus MW2 | NC_003923 |
| Staphylococcus aureus subsp. aureus Mu3 | NC_009782 |
| Staphylococcus aureus subsp. aureus Mu50 | NC_002758 |
| Staphylococcus aureus subsp. aureus N315 | NC_002745 |
| Staphylococcus aureus subsp. aureus NCTC 8325 | NC_007795 |
| Staphylococcus aureus subsp. aureus USA300 | NC_007793 |
| Staphylococcus aureus subsp. aureus USA300_TCH1516 | NC_010079 |
| Staphylococcus aureus subsp. aureus str. Newman | NC_009641 |
| Staphylococcus epidermidis ATCC 12228 | NC_004461 |
| Staphylococcus epidermidis RP62A | NC_002976 |
| Staphylococcus haemolyticus JCSC1435 | NC_007168 |
| Staphylococcus saprophyticus subsp. saprophyticus ATCC 15305 | NC_007350 |
| Streptococcus agalactiae 2603V/R | NC_004116 |
| Streptococcus agalactiae A909 | NC_007432 |
| Streptococcus agalactiae NEM316 | NC_004368 |
| Streptococcus gordonii str. Challis substr. CH1 | NC_009785 |
| Streptococcus mutans UA159 | NC_004350 |
| Streptococcus pneumoniae D39 | NC_008533 |
| Streptococcus pneumoniae R6 | NC_003098 |
| Streptococcus pneumoniae TIGR4 | NC_003028 |
| Streptococcus pyogenes M1 GAS | NC_002737 |
| Streptococcus pyogenes MGAS10270 | NC_008022 |
| Streptococcus pyogenes MGAS10394 | NC_006086 |
| Streptococcus pyogenes MGAS10750 | NC_008024 |
| Streptococcus pyogenes MGAS2096 | NC_008023 |
| Streptococcus pyogenes MGAS315 | NC_004070 |
| Streptococcus pyogenes MGAS5005 | NC_007297 |
| Streptococcus pyogenes MGAS6180 | NC_007296 |
| Streptococcus pyogenes MGAS8232 | NC_003485 |
| Streptococcus pyogenes MGAS9429 | NC_008021 |
| Streptococcus pyogenes SSI-1 | NC_004606 |
| Streptococcus pyogenes str. Manfredo | NC_009332 |
| Streptococcus sanguinis SK36 | NC_009009 |
| Streptococcus suis 05ZYH33 | NC_009442 |
| Streptococcus suis 98HAH33 | NC_009443 |
| Streptococcus thermophilus CNRZ1066 | NC_006449 |
| Streptococcus thermophilus LMD-9 | NC_008532 |
| Streptococcus thermophilus LMG 18311 | NC_006448 |
| Streptomyces avermitilis MA-4680 | NC_003155 |
| Streptomyces coelicolor A3(2) | NC_003888 |
| Sulfurimonas denitrificans DSM 1251 | NC_007575 |
| Sulfurovum sp. NBC37-1 | NC_009663 |
| Symbiobacterium thermophilum IAM 14863 | NC_006177 |
| Synechococcus elongatus PCC 6301 | NC_006576 |
| Synechococcus elongatus PCC 7942 | NC_007604 |
| Synechococcus sp. CC9311 | NC_008319 |
| Synechococcus sp. CC9605 | NC_007516 |
| Synechococcus sp. CC9902 | NC_007513 |
| Synechococcus sp. JA-2-3B'a(2-13) | NC_007776 |
| Synechococcus sp. JA-3-3Ab | NC_007775 |
| Synechococcus sp. RCC307 | NC_009482 |
| Synechococcus sp. WH 7803 | NC_009481 |
| Synechococcus sp. WH 8102 | NC_005070 |
| Synechocystis sp. PCC 6803 | NC_000911 |
| Syntrophobacter fumaroxidans MPOB | NC_008554 |
| Syntrophomonas wolfei subsp. wolfei str. Goettingen | NC_008346 |
| Syntrophus aciditrophicus SB | NC_007759 |
| Thermoanaerobacter pseudethanolicus ATCC 33223 | NC_010321 |
| Thermoanaerobacter sp. X514 | NC_010320 |
| Thermoanaerobacter tengcongensis MB4 | NC_003869 |
| Thermobifida fusca YX | NC_007333 |
| Thermosipho melanesiensis BI429 | NC_009616 |
| Thermosynechococcus elongatus BP-1 | NC_004113 |
| Thermotoga lettingae TMO | NC_009828 |
| Thermotoga maritima MSB8 | NC_000853 |
| Thermotoga petrophila RKU-1 | NC_009486 |
| Thermus thermophilus HB27 | NC_005835 |
| Thermus thermophilus HB8 | NC_006461 |
| Thiobacillus denitrificans ATCC 25259 | NC_007404 |
| Thiomicrospira crunogena XCL-2 | NC_007520 |
| Treponema denticola ATCC 35405 | NC_002967 |
| Treponema pallidum subsp. pallidum str. Nichols | NC_000919 |
| Trichodesmium erythraeum IMS101 | NC_008312 |
| Tropheryma whipplei TW08/27 | NC_004551 |
| Tropheryma whipplei str. Twist | NC_004572 |
| Ureaplasma parvum serovar 3 str. ATCC 700970 | NC_002162 |
| Verminephrobacter eiseniae EF01-2 | NC_008786 |
| Vibrio cholerae O1 biovar eltor str. N16961 | NC_002506 NC_002505 |
| Vibrio cholerae O395 | NC_009456 NC_009457 |
| Vibrio fischeri ES114 | NC_006840 NC_006841 |
| Vibrio harveyi ATCC BAA-1116 | NC_009784 NC_009783 |
| Vibrio parahaemolyticus RIMD 2210633 | NC_004603 NC_004605 |
| Vibrio vulnificus CMCP6 | NC_004460 NC_004459 |
| Vibrio vulnificus YJ016 | NC_005139 NC_005140 |
| Wigglesworthia glossinidia endosymbiont of Glossina brevipalpis | NC_004344 |
| Wolbachia endosymbiont of Drosophila melanogaster | NC_002978 |
| Wolbachia endosymbiont strain TRS of Brugia malayi | NC_006833 |
| Wolinella succinogenes DSM 1740 | NC_005090 |
| Xanthobacter autotrophicus Py2 | NC_009720 |
| Xanthomonas axonopodis pv. citri str. 306 | NC_003919 |
| Xanthomonas campestris pv. campestris str. 8004 | NC_007086 |
| Xanthomonas campestris pv. campestris str. ATCC 33913 | NC_003902 |
| Xanthomonas campestris pv. vesicatoria str. 85-10 | NC_007508 |
| Xanthomonas oryzae pv. oryzae KACC10331 | NC_006834 |
| Xanthomonas oryzae pv. oryzae MAFF 311018 | NC_007705 |
| Xylella fastidiosa 9a5c | NC_002488 |
| Xylella fastidiosa Temecula1 | NC_004556 |
| Yersinia enterocolitica subsp. enterocolitica 8081 | NC_008800 |
| Yersinia pestis Angola | NC_010159 |
| Yersinia pestis Antiqua | NC_008150 |
| Yersinia pestis CO92 | NC_003143 |
| Yersinia pestis KIM | NC_004088 |
| Yersinia pestis Nepal516 | NC_008149 |
| Yersinia pestis Pestoides F | NC_009381 |
| Yersinia pestis biovar Microtus str. 91001 | NC_005810 |
| Yersinia pseudotuberculosis IP 31758 | NC_009708 |
| Yersinia pseudotuberculosis IP 32953 | NC_006155 |
| Zymomonas mobilis subsp. mobilis ZM4 | NC_006526 |
