# Supplementary material for: A simple, fast, and accurate method of phylogenomic inference
Source: Genome Biol. 2008 Oct 13;9(10):R151. doi: 10.1186/gb-2008-9-10-r151 (PMC2760878; doi:10.1186/gb-2008-9-10-r151)
Supplement: Additional data file 5 — Presented is a table listing phylotypes breakdown of the Sargasso Sea metagenomic sequence data by phylogenetic markers and major taxonomic groups. [file gb-2008-9-10-r151-S5.doc]

| **Marker** | Alphaproteobacteria | Betaproteobacteria | Gammaproteobacteria | Deltaproteobacteria | Epsilonproteobacteria | Unclassified Proteobacteria | Bacteroidetes | Chlamydiae | Cyanobacteria | Acidobacteria | Thermotogae | Fusobacteria | Actinobacteria | Aquificae | Planctomycetes | Spirochaetes | Firmicutes | Chloroflexi | Chlorobi | Unclassified Bacteria | **SUM** |
| --- | --- | --- | --- | --- | --- | --- | --- | --- | --- | --- | --- | --- | --- | --- | --- | --- | --- | --- | --- | --- | --- |
| dnaG | 488 | 5 | 137 | 4 |  | 3 | 21 | 1 | 34 |  | 6 |  |  |  | 1 | 10 | 34 | 2 |  | 45 | 791 |
| frr | 223 | 3 | 64 | 5 |  |  | 27 | 1 | 29 | 2 |  |  | 1 | 2 | 2 | 37 | 4 | 3 |  | 37 | 440 |
| infC | 385 | 5 | 61 | 9 |  | 2 | 21 |  | 16 | 1 | 1 |  | 10 |  | 2 | 7 | 2 | 2 |  | 31 | 555 |
| nusA | 613 | 5 | 157 | 12 |  | 14 | 12 |  | 36 |  |  |  | 4 |  |  | 3 | 8 | 7 |  | 72 | 943 |
| pgk | 560 | 3 | 56 | 15 |  | 52 | 27 | 6 | 35 |  |  |  | 10 |  | 6 | 17 | 30 | 2 |  | 19 | 838 |
| pyrG | 511 | 7 | 175 | 13 | 1 | 6 | 29 |  | 41 |  | 2 |  | 7 | 2 | 3 | 31 | 21 | 4 |  | 45 | 898 |
| rplA | 345 | 4 | 97 | 6 |  | 8 | 24 |  | 41 |  | 4 |  | 5 |  | 5 | 1 | 7 | 8 |  | 31 | 586 |
| rplB | 389 | 4 | 81 | 19 |  | 29 | 17 |  | 29 | 6 |  |  | 6 | 1 | 1 | 5 | 2 | 4 |  | 46 | 639 |
| rplC | 304 | 3 | 87 | 9 |  | 13 | 26 |  | 30 |  |  |  | 1 |  | 2 | 13 | 3 | 3 | 2 | 29 | 525 |
| rplD | 308 | 2 | 91 | 2 |  | 21 | 21 |  | 23 |  |  |  | 4 |  | 1 |  | 5 | 1 |  | 35 | 514 |
| rplE | 319 | 5 | 101 | 4 |  | 5 | 22 |  | 28 |  |  | 1 | 3 |  |  | 1 | 6 | 7 |  | 18 | 520 |
| rplF | 329 | 8 | 81 | 13 | 1 | 24 | 17 | 1 | 29 |  | 1 |  | 5 | 2 |  |  | 8 |  |  | 42 | 561 |
| rplK | 314 | 6 | 40 | 6 | 1 | 2 | 25 | 3 | 28 |  | 1 |  | 7 |  | 2 | 10 | 3 |  | 5 | 41 | 494 |
| rplL | 289 | 4 | 69 | 25 | 1 | 12 | 26 |  | 26 |  |  |  | 9 |  | 1 | 4 |  | 1 |  | 14 | 481 |
| rplM | 260 | 5 | 28 | 8 |  | 3 | 23 |  | 30 |  | 3 |  | 3 |  |  | 10 | 4 | 8 |  | 62 | 447 |
| rplN | 288 | 5 | 57 | 2 |  | 25 | 17 |  | 23 | 4 |  |  | 10 | 1 | 1 | 1 | 12 | 1 | 1 | 73 | 521 |
| rplP | 299 | 3 | 70 | 9 |  | 20 | 18 |  | 19 |  |  |  | 10 |  | 2 | 5 | 1 | 5 |  | 19 | 480 |
| rplS | 282 | 2 | 47 | 3 |  | 26 | 20 |  | 16 |  | 2 |  | 4 |  | 2 | 5 |  | 12 |  | 28 | 449 |
| rplT | 314 | 4 | 53 | 8 |  | 13 | 18 | 1 | 34 |  | 1 | 1 |  |  | 1 | 2 | 6 | 2 |  | 56 | 514 |
| rpmA | 226 | 17 | 81 | 6 |  | 1 | 16 |  | 18 |  | 2 |  | 1 |  | 1 | 16 | 4 |  |  | 17 | 406 |
| rpoB | 1329 | 10 | 334 | 17 |  | 29 | 70 | 1 | 114 |  |  |  | 28 |  | 5 | 2 | 4 | 18 | 2 | 137 | 2100 |
| rpsB | 285 | 2 | 87 | 3 |  | 10 | 23 |  | 33 |  | 1 |  |  |  | 1 | 3 | 40 | 7 |  | 41 | 536 |
| rpsC | 348 | 5 | 67 | 4 |  | 16 | 31 |  | 20 | 1 |  |  | 10 | 2 | 1 | 1 | 4 |  | 1 | 53 | 564 |
| rpsE | 325 | 3 | 80 | 2 |  | 8 | 18 |  | 18 |  |  |  |  |  | 1 | 1 | 3 | 17 |  | 32 | 508 |
| rpsI | 225 | 3 | 65 | 7 | 1 | 18 | 18 |  | 28 | 5 |  |  | 4 |  |  | 5 | 2 | 1 |  | 35 | 417 |
| rpsJ | 272 | 2 | 51 | 7 |  | 25 | 19 |  | 29 |  |  |  | 2 |  | 2 |  | 5 |  |  | 44 | 458 |
| rpsK | 275 | 4 | 71 | 18 |  | 26 | 20 | 1 | 26 |  | 3 |  | 3 |  | 1 | 3 | 2 |  | 1 | 30 | 484 |
| rpsM | 314 | 5 | 69 | 15 | 1 | 12 | 21 |  | 28 | 1 | 8 |  | 9 |  | 2 | 4 | 4 | 1 |  | 22 | 516 |
| rpsS | 224 | 2 | 83 | 11 |  |  | 24 |  | 16 | 1 |  |  | 4 |  | 3 | 2 | 2 | 1 |  | 44 | 417 |
| smpB | 253 | 2 | 56 | 4 |  | 6 | 15 |  | 27 |  | 1 |  | 4 |  |  | 2 | 21 |  | 6 | 42 | 439 |
| tsf | 289 | 6 | 98 | 25 | 3 | 16 | 22 | 2 | 21 |  | 4 |  | 6 |  |  | 18 | 15 | 3 |  | 38 | 566 |
| **SUM** | 11185 | 144 | 2694 | 291 | 9 | 445 | 708 | 17 | 925 | 21 | 40 | 2 | 170 | 10 | 49 | 219 | 262 | 120 | 18 | 1278 | 18607 |
